# A High-Fat Meal, or Intraperitoneal Administration of a Fat Emulsion, Increases Extracellular Dopamine in the Nucleus Accumbens

**DOI:** 10.3390/brainsci2020242

**Published:** 2012-06-11

**Authors:** Pedro Rada, Nicole M. Avena, Jessica R. Barson, Bartley G. Hoebel, Sarah F. Leibowitz

**Affiliations:** 1 Laboratory of Behavioral Physiology, School of Medicine, University of Los Andes, Mérida 5101-A, Venezuela; Email: radap@ula.ve; 2 Department of Psychology and Princeton Neuroscience Institute, Princeton University, Princeton, NJ 08544, USA; 3 Department of Psychiatry, College of Medicine, University of Florida, Gainesville, FL 32610, USA; Email: navena@ufl.edu; 4 Laboratory of Behavioral Neurobiology, The Rockefeller University, New York, NY 10065, USA; Email: jbarson@rockefeller.edu

**Keywords:** dopamine, nucleus accumbens, triglycerides, high-fat, microdialysis, rat

## Abstract

Evidence links dopamine (DA) in the nucleus accumbens (NAc) shell to the ingestion of palatable diets. Less is known, however, about the specific relation of DA to dietary fat and circulating triglycerides (TG), which are stimulated by fat intake and promote overeating. The present experiments tested in Sprague-Dawley rats whether extracellular levels of NAc DA increase in response to acute access to fat-rich food or peripheral injection of a fat emulsion and, if so, whether this is related to caloric intake or elevated circulating lipids. When rats consumed more calories of a high-fat meal compared with a low-fat meal, there was a significant increase in extracellular accumbens DA (155% *vs.* 119%). Systemic injection of a fat emulsion, which like a high-fat diet raises circulating TG but eliminates the factor of taste and allows for the control of caloric intake, also significantly increased extracellular levels of DA (127%) compared to an equicaloric glucose solution (70%) and saline (85%). Together, this suggests that a rise in circulating TG may contribute to the stimulatory effect of a high-fat diet on NAc DA.

## 1. Introduction

Laboratory animals and humans tend to overeat when offered a fat-rich diet. This phenomenon may be mediated, in part, by a subset of orexigenic peptides, including the opioids, galanin and orexin [1]. Hypothalamic injection of these peptides stimulates the ingestion of a high- *vs.* low-fat diet [2,3,4], and consumption of a high-fat diet stimulates hypothalamic expression and synthesis of these peptides [5,6,7]. This suggests a positive feedback loop between dietary fat and orexigenic peptides that contributes to the increased caloric intake induced by a fat-rich diet [8]. Also, circulating lipids, known to be elevated by a high-fat diet, may be involved in this positive feedback loop; injection of a fat emulsion that raises circulating levels of triglycerides (TG) promotes hyperphagia and stimulates the expression of orexigenic peptides [5,8] as well as the activity of neurons that produce them [9]. 

Dopamine (DA) has a diverse role in the reinforcement of various appetitive behaviors [10,11,12]. Dopamine may underlie incentive salience [13], act as an error signal between predicted and obtained rewards [14], or potentiate responsiveness and facilitate learning and flexibility of behavior [15]. In the nucleus accumbens (NAc), DA has a function in the motivation to seek and eat food [16]. Palatable tastes facilitate the intake of food, which in turn further stimulates DA release [17,18,19,20], and this effect is enhanced when animals are food deprived [21,22]. Hypothalamic injections of galanin or opioids, which stimulate consumption of a high-fat diet, increase NAc DA, suggesting that they may act, in part, through DA to produce their behavioral effects [23,24].

A fat-rich diet has a stimulatory effect on mesolimbic DA [25,26,27,28], but it is not known whether this is due to caloric load or the diet-induced increase in circulating lipids (e.g., TG). The possibility that DA may be related to the taste of fat receives support from evidence showing increased extracellular NAc DA in rats sham fed corn oil [27]; however, this effect is not specific to fat since NAc DA release is increased by drinking sucrose [17,29], and thus, may be related to palatability.

## 2. Results

### 2.1. Experiment 1: A High-Fat Meal Increases Extracellular NAc DA Levels More than a Less Palatable Low-Fat Meal

**Figure 1:**
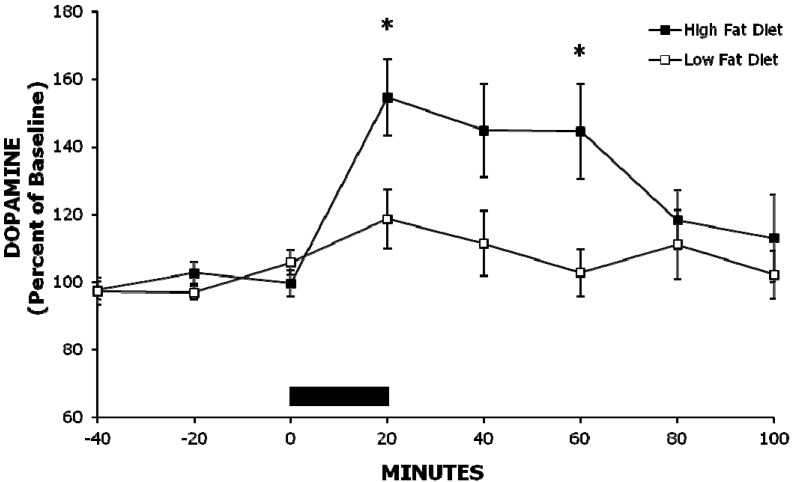
Consumption of a high-fat meal stimulates the release of accumbens dopamine more than consumption of a low-fat meal. While rats had the meal available for 60 min, they consumed most of it during the first 20 min of access (represented by the black bar on the ordinate). Mean ± SEM, * p < 0.05 *vs.* low-fat meal.

In order to test the effects of a high-fat *vs.* a low-fat meal on extracellular levels of DA in the NAc, we offered Sprague-Dawley rats acute (60 min) access to these diets, and then used *in vivo* microdialysis to assess the levels of DA in response to eating the meal. Rats consumed more when offered the high-fat meal compared with the low-fat meal (27.8 ± 1.7 *vs.* 13.9 ± 1.9 kcal, *p* < 0.001). The high-fat meal increased extracellular levels of NAc DA significantly more than the low-fat meal (*F* (1, 8) = 8.31, *p* < 0.05), and this effect was moderated by time (*F* (7, 56) = 2.95, *p* < 0.05; see Figure 1). Consumption of the high-fat meal also elevated DA release compared to baseline (*F* (7, 56) = 6.28, *p* < 0.001), with levels peaking to 155% of baseline at 20 min after meal presentation (*p* < 0.01; see Figure 1). This is in contrast to the low-fat meal, which compared to baseline did not significantly stimulate extracellular DA levels. 

DA metabolite data are shown in Table 1. Measurements of the metabolites revealed an increase in levels with both the high-fat and low-fat meals. Direct comparison between the high-fat and low-fat meals showed no significant difference in their stimulation of 3,4-dihydroxyphenylacetic acid (DOPAC) levels between the two groups. Levels of DOPAC were increased after the high-fat meal (*F* (7, 56) = 9.09, *p* < 0.001) and the low-fat meal (*F* (7, 56) = 5.51, *p* < 0.001). Homovanillic acid (HVA) was significantly elevated after both the high-fat meal (*F* (7, 56) = 7.97, *p* < 0.001) and the low-fat meal (*F* (7, 56) = 5.66, *p* < 0.001). 

**Table 1:** Percentages of baseline measurements of 3,4-dihydroxyphenylacetic acid (DOPAC) and homovanillic acid (HVA) according to time and condition.

**Experiment 1**	**Baseline**	**20 min**	**40 min**	**60 min**
**DOPAC**				
High-Fat Meal	99 ± 2%	124 ± 4%	125 ± 5%	122 ± 7%
Low-Fat Meal	102 ± 1%	113 ± 5%	115 ± 5%	112 ± 6%
**HVA**				
High-Fat Meal	102 ± 3%	117 ± 4%	126 ± 4%	130 ± 7%
Low-Fat Meal	100 ± 2%	112 ± 5%	119 ± 6%	120 ± 6%
**Experiment 2**	**Baseline**	**60 min**	**120 min**	**180 min**
**DOPAC**				
Intralipid	99 ± 1%	112 ± 7%	100 ± 5%	98 ± 3%
Glucose	97 ± 1%	93 ± 5%	86 ± 3%	82 ± 6%
Saline	101 ± 2%	94 ± 5%	108 ± 12%	103 ± 10%
**HVA**				
Intralipid	99 ± 2%	113 ± 8%	103 ± 4%	101 ± 5%
Glucose	98 ± 1%	95 ± 6%	90 ± 2%	92 ± 3%
Saline	98 ± 3%	97 ± 6%	105 ± 8%	103 ± 7%

### 2.2. Experiment 2: Injection of Intralipid Increases Extracellular NAc DA Levels Compared to Glucose or Saline

**Figure 2:**
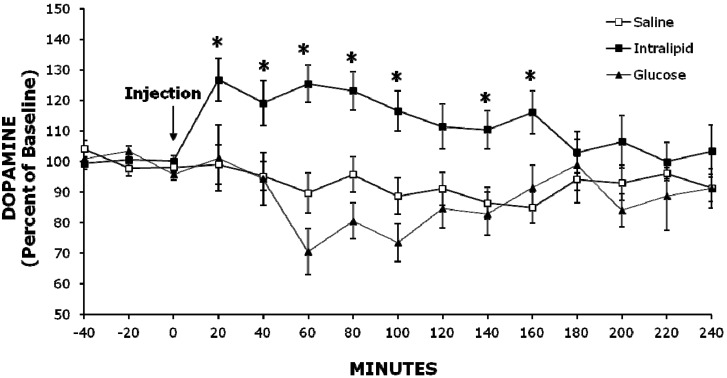
Injection of the fat emulsion Intralipid increases extracellular DA in the accumbens more than injection of saline or an equicaloric glucose solution. Mean ± SEM, * p < 0.05 *vs.* saline.

We next tested whether systemic injection of a fat emulsion (Intralipid), which causes a rise in circulating TG levels, would affect extracellular levels of DA in the NAc. This experiment allowed for assessment of the effects of increasing TG levels independent of taste and palatability factors. Injections of Intralipid, glucose or saline had different effects on extracellular NAc DA levels (*F* (2, 18) = 8.12, *p* < 0.01) that changed over time (*F* (28, 252) = 3.47, *p* < 0.001; see Figure 2). Specifically, Intralipid stimulated extracellular DA levels more than glucose between 40 min (+25%, *p* < 0.05) and 160 min (+25%, *p* < 0.05) post-injection, with the largest difference occurring at 60 min (+55%, *p* < 0.001). In addition, Intralipid increased extracellular DA more than saline between 20 min (+28%, *p* < 0.05) and 160 min (+31%, *p* < 0.05) post-injection, with the largest difference also at 60 min (+36%, *p* < 0.01). Intralipid increased extracellular DA compared to baseline (*F* (14, 112) = 3.83, *p* < 0.001) up to 127% at 20 min (*p* < 0.05) and 60 min (*p* < 0.05; see Figure 2). Glucose injection reduced extracellular DA levels (*F* (14, 70) = 3.08, *p* < 0.01; a nadir of 70% at 60 min), and the saline injection had no significant effect on DA. 

DA metabolite data are shown in Table 1. The DA metabolite levels following these injections reflect the release of DA. Injections had significant, overall effects on levels of DOPAC (*F* (2, 18) = 3.54, *p* < 0.05). Intralipid significantly enhanced extracellular levels of DOPAC (*F* (14, 112) = 2.08, *p* < 0.05), while glucose reduced DOPAC (*F* (14, 70) = 5.60, *p* < 0.001). Saline did not affect DOPAC levels. Neither Intralipid nor saline significantly affected HVA levels compared to baseline. However, glucose suppressed HVA levels (*F* (14, 70) = 3.28, *p* < 0.001). 

TG were significantly increased by Intralipid compared to glucose and saline (*F* (2, 12) = 39.63, *p* < 0.001). *Post hoc* tests showed that this difference was due to elevated TG levels after Intralipid compared to both glucose and saline (*p* < 0.001), with no differences between glucose and saline themselves. Injection of Intralipid increased TG levels compared to baseline (*F* (3, 12) = 8.18, *p* < 0.01), at 60 min (+98%), 120 min (+122%), and 240 min (+110%, *p* < 0.01) post injection. However, levels of TG were no different after injection of glucose or saline compared to baseline.

### 2.3. Histology

**Figure 3:**
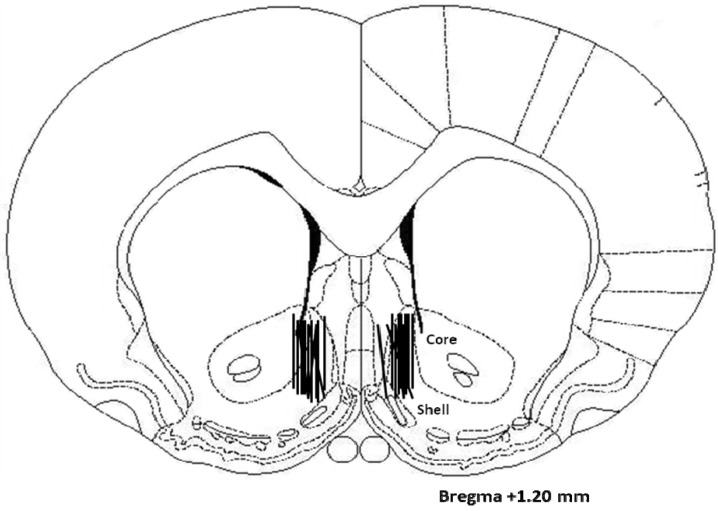
Microdialysis probes were located in the medial NAc shell.

Microdialysis probes were located predominately in the medial accumbens shell (see Figure 3). 

## 3. Discussion

A high-fat meal can stimulate the release of NAc DA, and while calories and palatability are factors believed to be involved, this effect can still occur even if these variables are controlled. Experiment 1 supports the existing literature by showing an increase in DA release after a fat-rich meal, while Experiment 2 demonstrates a similar effect with a fat emulsion, which bypasses palatability as a factor and controls for calorie load, supporting the involvement of post-ingestional factors. Collectively, these results demonstrate that a high-fat source itself, independent of calories or taste, can release NAc DA, and that this may be due to the central actions of fat metabolites, such as TG.

### 3.1. Role of Calories in Fat-Induced Increase in Extracellular Levels of NAc DA and Hyperphagia

A high-fat meal, compared to a meal of rodent chow or water, can increase the release of NAc DA [27,30], and Experiment 1 supports this finding by demonstrating an increase in extracellular DA following a high- *vs.* low-fat meal of similar macronutrient constituents. NAc DA was elevated to 155% of baseline right after the meal and then for another 40 min when food was available but little eating occurred. This magnitude of increase in DA release is biologically significant as it is within the range seen when animals engage in other appetitive behaviors, such as when they are fed palatable food [19,30] or are administered a drug of abuse, such as morphine [31]. Further, this prolonged increase in extracellular DA extended toward the time when the next meal should naturally occur [32] and might contribute to the larger meal that occurs following a high- *vs.* low-fat meal [8]. The finding that the low-fat meal did not increase extracellular levels of DA in the NAc is consistent with previous studies that suggest that DA release attenuates when a palatable food is no longer novel [19,30]. The fact that DA continues to rise in response to the high fat meal, however, suggests that other mechanisms may be involved.

A high-fat diet is known to be obesogenic, in part, due to its greater caloric density relative to a low-fat diet. Indeed, the results of Experiment 1 show that the high-fat diet is associated with greater consumption relative to the low-fat diet, and this could be associated with the effect on DA. However, this characteristic of a high-fat diet is not essential for eliciting hyperphagia, which can occur with diets equal in caloric density [8]. The results of Experiment 2 further support the idea that accumbens DA can be released independent of calories. This is evident with peripheral administration of the 50% glucose solution, which provided calories but did not increase DA release when compared to saline or Intralipid. Intragastric glucose is known to stimulate accumbens DA release [33]. However, the relatively high concentration of glucose in the present study, used in order to equal the calories of Intralipid, coupled with the route of administration, may have affected other brain systems that contributed to the decrease in DA release.

### 3.2. Role of Palatability in Fat-Induced Increase in Extracellular NAc DA Levels and Hyperphagia

Palatability and orosensory stimulation are important factors in stimulating DA. In addition to fat, access to the sweet carbohydrate, sucrose, increases the release of NAc DA [17,29,33] and this effect habituates with repeated exposure [30], but is sustained when the food is offered in a binge-like manner that promotes addiction-like behaviors [34]. While not directly measured, it is presumed that the high-fat diet in Experiment 1 was more palatable than the low-fat diet, as evidenced by the increase in consumption. Experiment 2, however, suggests that palatability is not an essential factor in the stimulatory effect of fat on DA release, just as it is not necessary for fat-induced hyperphagia [8]. Extracellular DA was markedly increased by Intralipid injections, which bypassed the effects of orosensory cues. 

### 3.3. Role of Circulating Lipids in Fat-Induced Increases in Extracellular NAc DA Levels and Hyperphagia

Circulating TG rise after consumption or administration of fat. Previous studies show that the rise in TG is consistently large and robust (100%–150%) after a small high-fat meal (10–15 kcal), much like the effect seen after larger meals [1,35]. This rise in TG is closely linked to hyperphagia that consistently follows an ingested or injected high-fat solution [8]. Peripheral injection of Intralipid produced an increase in extracellular DA, and this effect was evident when compared to equicaloric glucose as well as to saline. While there is little evidence directly linking circulating lipids to accumbens DA, one study shows that a deficiency of fatty acids in the diet, which lowers circulating lipids, causes a reduction in mesolimbic DA activity [36]. In addition, fat delivered intragastrically to avoid taste can nonetheless act as a reinforcer in a conditioning paradigm [37], suggesting that taste conditioning depends, in part, on stimulation of accumbens DA [38]. 

## 4. Experimental Procedure

### 4.1. Subjects

Male, Sprague-Dawley rats (approximately 300 g) were obtained from Taconic Farms (Germantown, NY) and housed individually in the Princeton University vivarium on a reversed 12 h light/12 h dark cycle. All rats were maintained *ad libitum* on rodent chow (LabDiet #5001, PMI Nutrition International, Richmond, IN) and water, except as indicated in the experimental design. All procedures were conducted in accordance with the Princeton University Institutional Animal Care and Use Committee and conformed to the National Institutes of Health guidelines on the ethical use of animals. 

### 4.2. Surgery

After at least one week of acclimation to the vivarium, rats underwent surgery to implant guide cannulas for microdialysis. They were anesthetized with 10 mg/kg xylazine and 100 mg/kg ketamine (i.p.), supplemented with ketamine as needed. Using a stereotaxic instrument, bilateral 21 gauge stainless-steel guide cannulas were implanted and aimed at the posterior medial accumbens shell (+1.2 mm anterior, 0.8 mm lateral and 4.0 mm ventral, 5 mm above the NAc, with reference to bregma, midsagittal sinus, and surface of the level skull, respectively). 

### 4.3. Microdialysis and DA Assays

Approximately one week after surgery, a microdialysis probe was inserted and cemented in place, protruding 9 mm ventral to the skull surface to reach the intended site in the NAc shell. Probes were fixed in place at least 16 h before the microdialysis session to allow neurotransmitter recovery to stabilize. Microdialysis probes were constructed as described in an earlier report [39]. Probes were perfused with buffered Ringer’s solution (142 mM NaCl, 3.9 mM KCl, 1.2 mM CaCl_2_, 1.0 mM MgCl_2_, 1.35 mM Na_2_HPO_4_, 0.3 mM NaH_2_PO_4_, pH 7.3) at a flow rate of 0.5 µL/min. The flow rate was increased to 1.0 μL/min 2 h before and throughout the microdialysis session.

Extracellular DA and its metabolites, DOPAC and HVA, were analyzed by reverse phase, high-performance liquid chromatography with electrochemical detection, as described in an earlier report [14]. 

### 4.4. Diets

In Experiment 1, diets were presented to the rats in round glass jars. As previously described [40], the solid high-fat diet (5.15 kcal/g) contained 50% fat (82% lard and 18% vegetable oil), 25% carbohydrate (30% dextrin, 30% cornstarch and 40% sucrose) and 25% protein (100% casein). The low-fat diet (3.93 kcal/g) contained 10% fat (100% vegetable oil), 65% carbohydrate (15% dextrin, 15% cornstarch and 70% sucrose) and 25% protein (100% casein). 

### 4.5. Test Procedures

*Experiment 1*: To test whether solid high-fat *vs.* low-fat meals differentially affect extracellular levels of NAc DA, rats (*N* = 9) were acclimated to these diets by removing their rodent chow for 4 h starting at dark onset and then offering 50 kcal of the high-fat or low-fat diet. Each diet was presented alone three times on alternating days, so that the rats were familiar with the taste of each diet. During this acclimation, the rats were allowed 30 min to consume the diets, after which their rodent chow was returned. Acclimation was done to ensure that the rats would not experience neophobia on the test day and to ensure that the effects on DA release were not related to the novelty of the diets. On the test day, the rats were food deprived for 14–16 h to ensure that they were not sated, and then 20-min microdialysis samples were collected starting 2 h into the dark phase before, during and after a 60-min presentation of either the high-fat or low-fat diet (30 kcal each). The presentation was counterbalanced, with each rat having a test with each diet on alternating days in a within-subject design. Baseline microdialysis samples were collected until DA levels were stable (an average of three samples), and they continued for 100 min following diet presentation. Water was removed immediately prior to sample collection. 

*Experiment 2*: This experiment was performed using systemic injection of fat to eliminate the effects of taste on extracellular NAc DA levels. Rats were given an i.p. injection (5 mL) of: (1) Intralipid (*n* = 9; 10 kcal, 20% fat emulsion, Baxter Healthcare Corporation, Deerfield, IL); (2) glucose (*n* = 6; 10 kcal, 50% solution); or (3) saline (*n* = 6). 20-min microdialysis samples were collected starting 2 h into the dark phase before, during and after the injections. Baseline microdialysis samples were collected until DA levels were stable (on average three samples), and sample collection continued for 240 min after injection. Food and water were removed immediately prior to sample collection, as deprivation to promote ingestion was not necessary.

*Measurements of TG*: To test whether levels of TG are differentially affected by the injection of Intralipid, glucose or saline, an additional set of rats was tested, using similar procedures as described above but with blood collections for TG measurements. These rats (*n* = 5/group), using a between-subject design, were given an i.p. injection (5 mL) of Intralipid (10 kcal), glucose (10 kcal) or saline, and tail vein blood was drawn for measurement of TG levels immediately prior to injection and 60, 120, and 240 min after injection. Serum from tail vein blood was assayed using a Triglyceride Assay kit (Sigma-Aldrich Co., St. Louis, MO). 

### 4.6. Histology

At the end of each microdialysis experiment, rats were overdosed with sodium pentobarbital. Brains were sectioned (40 µm) by a researcher blind to the experimental conditions in order to verify and plot microdialysis probe placement [41]. 

### 4.7. Data Analysis

Intake of diets was measured to the nearest 0.1 g and analyzed by paired *t*-tests. Microdialysis data were normalized to percent of the baseline samples. For Experiment 1, microdialysis data were analyzed by repeated measures ANOVA (with group and time as within-subject factors) followed by one-way repeated measures ANOVA (with time as within-subject factor) and Neuman-Keuls *post hoc* tests. For Experiment 2, microdialysis data were analyzed by repeated measures ANOVA (with group as the between-subject factor and time as the within-subject factor) followed by Tukey’s HSD and independent-samples *t*-tests and then by one-way repeated measures ANOVA and Neuman-Keuls *post hoc* tests. Data for TG were analyzed by repeated measures ANOVA (with group as the between-subject factor and time as the within-subject factor) followed by Tukey’s HSD and then by one-way repeated measures ANOVA, Neuman-Keuls *post hoc* tests and independent-samples *t*-tests. All values are expressed as mean ± standard error of the mean (SEM). 

## 5. Conclusion

These results demonstrate for the first time that, like ingestion, the injection of a high-fat solution that bypasses the mouth and gastrointestinal tract can cause an increase in extracellular accumbens DA. This finding underscores the importance of post-ingestional reward signals in high-fat feeding and their effects on innate and conditioned food intake.

## Abbreviations

DA: dopamine
DOPAC: 3,4-dihydroxyphenlyacetic acid
HVA: homovanillic acid
NAc: nucleus accumbens
TG: triglycerides
